# The randomized shortened dental arch study (RaSDA): design and protocol

**DOI:** 10.1186/1745-6215-11-15

**Published:** 2010-02-19

**Authors:** Ralph G Luthardt, Birgit Marré, Achim Heinecke, Joachim Gerss, Hans Aggstaller, Eckhard Busche, Paul Dressler, Ingrid Gitt, Wolfgang Hannak, Sinsa Hartmann, Guido Heydecke, Florentine Jahn, Matthias Kern, Torsten Mundt, Peter Pospiech, Helmut Stark, Bernd Wöstmann, Michael H Walter

**Affiliations:** 1 Clinic of Prosthetic Dentistry, Ulm University, University Hospital, Department of Dentistry, Ulm, Germany; 2 Department of Prosthetic Dentistry, Dresden University of Technology, University Hospital Carl Gustav Carus, Dental School, Dresden, Germany; 3 Coordinating Centre for Clinical Trials of the University, Münster, Germany; 4 Department of Medical Informatics and Biomathematics, University of Münster, Germany; 5 Department of Prosthetic Dentistry, Ludwig-Maximilians University Munich, Munich, Germany; 6 Department of Prosthetic Dentistry, Witten-Herdecke University, Witten, Germany; 7 Department of Prosthetic Dentistry, Julius-Maximilians University of Würzburg, Wurzburg, Germany; 8 Department of Prosthetic Dentistry, University of Leipzig, Leipzig, Germany; 9 Department of Prosthetic Dentistry, Charité Universitätsmedizin Berlin, Campus Benjamin Franklin, Berlin, Germany; 10 Department of Prosthetic Dentistry, Johannes-Gutenberg University of Mainz, Mainz, Germany; 11 Center for Dental and Oral Medicine, Department of Dental Prosthetics, University Medical Center Hamburg-Eppendorf, Hamburg, Germany; 12 Department of Prosthetic Dentistry, Friedrich-Schiller University of Jena, Florentine, Jena, Germany; 13 Department of Prosthetic Dentistry, Christan-Albrechts University of Kiel, Kiel, Germany; 14 Department of Prosthetic Dentistry, Ernst-Moritz-Arndt University of Greifswald, Greifswald, Germany; 15 Department of Prosthetic Dentistry, Saarland University Hospital and Saarland University Faculty of Medicine, Saarland, Germany; 16 Department of Prosthetic Dentistry, University of Bonn, Bonn, Germany; 17 Department of Prosthetic Dentistry, Justus-Liebig University of Giessen, Giessen, Germany

## Abstract

**Background:**

Various treatment options for the prosthetic treatment of jaws where all molars are lost are under discussion. Besides the placement of implants, two main treatment types can be distinguished: replacement of the missing molars with removable dental prostheses and non-replacement of the molars, i.e. preservation of the shortened dental arch. Evidence is lacking regarding the long-term outcome and the clinical performance of these approaches. High treatment costs and the long time required for the treatment impede respective clinical trials.

**Methods/design:**

This 14-center randomized controlled investigator-initiated trial is ongoing. Last patient out will be in 2010. Patients over 35 years of age with all molars missing in one jaw and with at least both canines and one premolar left on each side were eligible. One group received a treatment with removable dental prostheses for molar replacement (treatment A). The other group received a treatment limited to the replacement of all missing anterior and premolar teeth using fixed bridges (treatment B). A pilot trial with 32 patients was carried out. Two hundred and fifteen patients were enrolled in the main trial where 109 patients were randomized for treatment A and 106 for treatment B. The primary outcome measure is further tooth loss during the 5-year follow-up. The secondary outcome measures encompassed clinical, technical and subjective variables. The study is funded by the Deutsche Forschungsgemeinschaft (German Research Foundation, DFG WA 831/2-1, 2-2, 2-3, 2-4, 2-5).

**Discussion:**

The particular value of this trial is the adaptation of common design components to the very specific features of complex dental prosthetic treatments. The pilot trial proved to be indispensable because it led to a number of adjustments in the study protocol that considerably improved the practicability. The expected results are of high clinical relevance and will show the efficacy of two common treatment approaches in terms of oral health. An array of secondary outcome measures will deliver valuable supplementary information. If the results can be implemented in the clinical practice, the daily dental care should strongly profit thereof.

**Trial registration:**

The trial is registered at ClinicalTrials.gov under ISRCTN68590603 (pilot trial) and ISRCTN97265367 (main trial).

## Background

Tooth loss is the consequence of the most common oral diseases caries and periodontitis [1-3]. It often starts with the first molar and, thus, results in tooth bounded spaces. Further losses often lead to shortened dental arches on either or both sides. A correlation between limited masticatory efficiency, restricted masticatory performance, and decreased patient satisfaction with a declining number of posterior teeth was found in several studies [4-6].

• no prosthetic treatment if the shortened dental arch is complete up to the second premolar

• the restoration of a shortened dental arch up to the second premolar with crowns and bridges on natural teeth or dental implants (no replacement of the molars)

• the replacement of missing molars with implant-retained crowns and bridges

• the replacement of missing molars with removable dental prostheses (RDPs) of various designs.

The minimum treatment with a preservation or restoration of the shortened dental arch was described by Käyser as the shortened dental arch (SDA) concept in 1981 [7]. This concept defined a dentition of a minimum of 10 occluding tooth pairs (e.g. all anterior teeth and premolars) as a suboptimal but still acceptable functional level. The SDA concept has been discussed controversially regarding side effects of non-replaced molars such as:

(1) higher rates of temporomandibular disorders

(2) tooth migration, overeruption and increased wear

(3) insufficient chewing efficiency and performance

(4) compromised aesthetics [8-11].

Missing teeth can be replaced using implant-retained restorations, tooth-borne bridges, and RDPs. Dental professionals consider the replacement of missing posterior teeth with implants a No. 1 choice especially with regard to the prevention of bone loss after tooth loss [12]. The access to implants, however, is limited by a number of factors, above all: high costs. The most serious complications in implant restorations are inflammatory processes and implant loss. Adverse effects of tooth-borne bridges include endodontic complications, tooth fracture, gingivitis, and secondary caries. In clinical trials dealing with bridges, both patients and professionals perceived a high benefit of it. Overall success rates of 90% for bridges after ten years of service are considerably high [13,14]. Less than 5% of the abutment teeth had to be removed within 10 years [14]. A superior performance of bridges compared to RDPs was reported in a long-term trial in a selected group of patients. In 52 patients (27 with bridges/25 with RDPs), 12 teeth had to be removed over a period of five years, 1 in the bridge group and 11 in the RDP group [15]. The treatment with RDPs seems to avoid adverse effects of missing molars and to improve patient satisfaction, masticatory efficiency as well as masticatory performance. However, it is known that patients with RDPs are compromised by a high incidence of side effects and complications such as increased plaque accumulation, high caries rates, and periodontal breakdown [16,17]. Furthermore, the benefit of RDPs for mere molar replacement has not been proven yet [18,19]. For the SDA concept with the non-replacement of molars, conclusive evidence for an increased occurrence of side effects has not been provided so far.

"The randomized shortened dental arch study" (RaSDA) was designed to evaluate the efficacy of the treatment with RDPs for molar replacement (treatment A) compared to the treatment limited to the replacement of all missing anterior and premolar teeth by means of fixed bridges (treatment B). Both treatment approaches chosen are considered as acceptable dental care. In many ways, shortened dental arches are borderline conditions in terms of the differential indication between fixed and removable restorations. Therefore, they are an ideal field to gather comparable data on the clinical performance of these fundamentally differing treatment concepts.

## Methods/design

This study was designed as a randomized multi-center trial. It will be reported according to the CONSORT statement [20]. The study was designed according to:

▪ World's Medical Association's Declaration of Helsinki

▪ International Organization for Standardization/European Standard (ISO/EN 540), Clinical Investigation of Medical Devices for Human Subjects, and

▪ Guidelines of Good Clinical Practice in Europe, July 1995

A consensus concerning all design aspects including treatment and dental laboratory procedures, basic dental materials, data collection, length, and frequency of the follow-ups was achieved and gathered in a detailed protocol. A manual describes the two treatment concepts in detail and is also available at the study homepage http://www.zahnreihe.de. The study design was approved by the Ethics Commission of the Medical Faculty Carl Gustav Carus of the Technical University Dresden (processing number: EK 260399).

### Pilot trial

The enrollment period for the pilot study was originally set to 6 months. A total of 78 patients should have been enrolled within this time. However, this target patient number could not be reached even after a prolongation. The enrollment phase for the pilot trial started in December 2000 and was finished in October 2001. Diagnostic measures commonly used in clinical dentistry were applied in the pilot study (Table 1). The analysis and the discussion of the information gathered during the enrollment phase for the pilot study took place at an international workshop held in October 2001 in Dresden. Based on the results and experience gained during the pilot study, conclusions were drawn and changes in the study design were made resulting in a slightly reduced number of outcome measures in the main trial (Table 1).

**Table 1:** Primary and secondary outcome measures.

Primary outcome measure		
**Oral/dental**	**Prosthetic**	**Subjective**

First tooth loss after prosthetic treatment		

**Secondary outcome measure**		

Second and further tooth loss	*Over-all performance*	*Oral health related quality of life*
	Breakdown of the treatment concept (need of complete renewal and change of treatment concept)	Oral health impact profile (OHIP): Measure of self-reported complaints attributed to oral conditions

*Tooth and pulp*	*Performance*	*Psyche*
Caries (crown/root) Abrasion Sensibility (pulp)	Marginal fit Loss of retention (crowns and bridges) Need of repair and relining Attachment performance* Technical complication Aesthetics rated by the dentist*	Psychological distress and dysfunction (Research Diagnostic Criteria for TMD [29,30])

*Periodontium*		
Plaque index [24] Probing depth/attachment loss/bleeding on probing (BOP)/furcation involvement Gingival index* [24] Tooth mobility		

*Function*		
Clinical dysfunction index [29] (Temporomandibular joint, muscles, movements, pain) Occlusion static/dynamic		

*Further*		
Interdental spacing in the anterior region Proximal contacts (shape/strength) Mucosa lesions Denture plaque Alveolar ridge resorption		

(*) excluded after pilot study

### Participants

The rationale behind the inclusion criteria chosen was to balance the chances for success. This assumption was based on clinical experience and a relatively sparse literature basis. However, any high-level evidence especially in terms of comparative trials is lacking.

#### Inclusion criteria in the main trial

**Figure 1:**
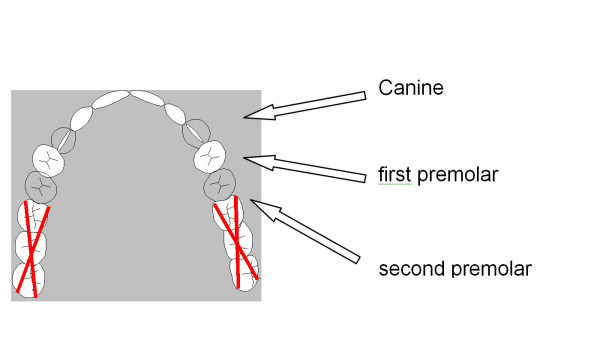
**Required topography in the jaw to be treated: Minimum dentition of both canines and one premolar per side and maximum dentition including all anterior teeth up to the second premolar on both sides**.

The patients had to meet the following inclusion criteria:

▪ all molars are missing in one jaw and at least both canines and one premolar are present on each side (Fig. 1)

▪ request of prosthetic treatment

▪ rejection of implant treatment

▪ over 35 years of age

▪ general health according to American Society of Anesthesiologists' classification group one or two [21]

#### Exclusion criteria in the main trial

Patients with any of the following conditions and attitudes were excluded:

▪ alcoholism and drug addiction

▪ psychic disorders

▪ acute signs and symptoms of temporomandibular disorders (TMD) or grade 2 of the anamnestic Helkimo Index [22,23]

▪ malocclusion of Angle class 2 or 3 with distal bite or mesial bite > one premolar width

▪ ongoing orthodontic treatment

▪ intention to undergo orthodontic treatment

▪ clinically acceptable prosthetic status

▪ rejection of RDPs

▪ request of complete molar replacement

▪ general health according to American Society of Anesthesiologists' (ASA) classification > 2 [21]

#### Settings and locations where the data was collected

Dental schools in Germany interested in clinical research were contacted describing the aims and the scope of the trial. Fourteen prosthetic departments of dental schools and one biometrical center joined the trial. The study team consists of the principle investigator, his deputy responsible for quality control, and the treatment coordinator. Every participating dental clinic appointed a local investigator and a dentist responsible for all treatment measures. The patients were recruited from the clientele of the according dental schools. All patients exhibiting a suitable dental status and requesting dental treatment were considered eligible for participation.

### Intervention

**Figure 2:**
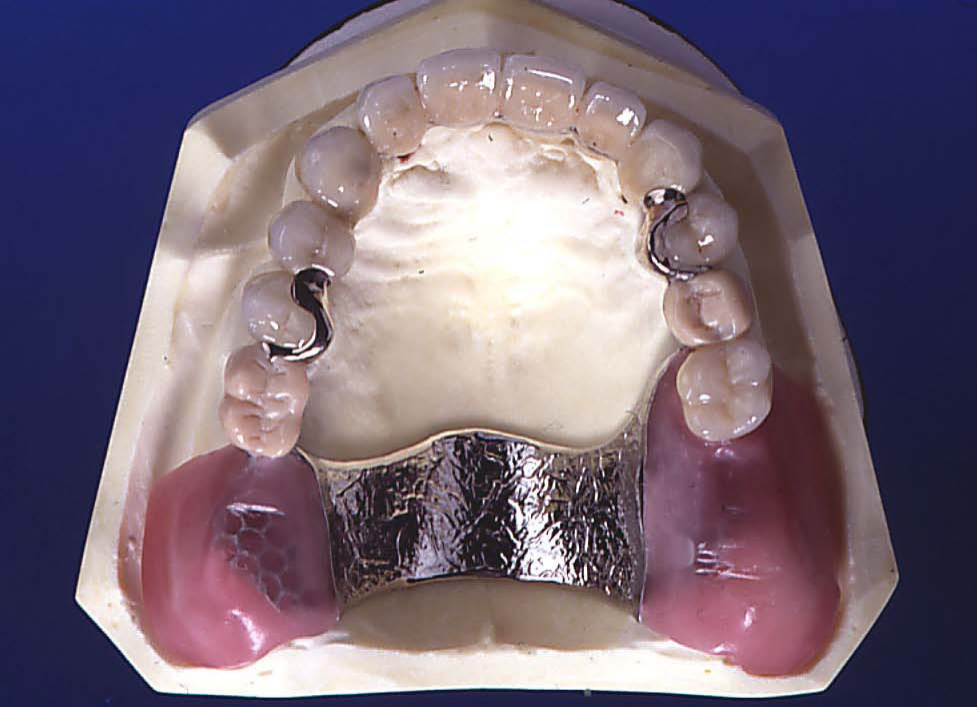
**Example of treatment A**.

Two treatment groups were defined:

Treatment A: Molars were replaced. This replacement was conducted by means of an RDP. A conventional cast framework retained by precision attachments (Mini SG No. 055 675) was supplemented with acrylic teeth. The male part of the attachment was part of a splinted crown or bridge retainer crown on the posterior-most tooth on each side. Retainer crowns were manufactured as porcelain fused to high noble metal crowns. If anterior teeth or first premolars were missing, they were replaced by bridges. If the second premolar was missing, this tooth was replaced by the RDP (Fig. 2).

**Figure 3:**
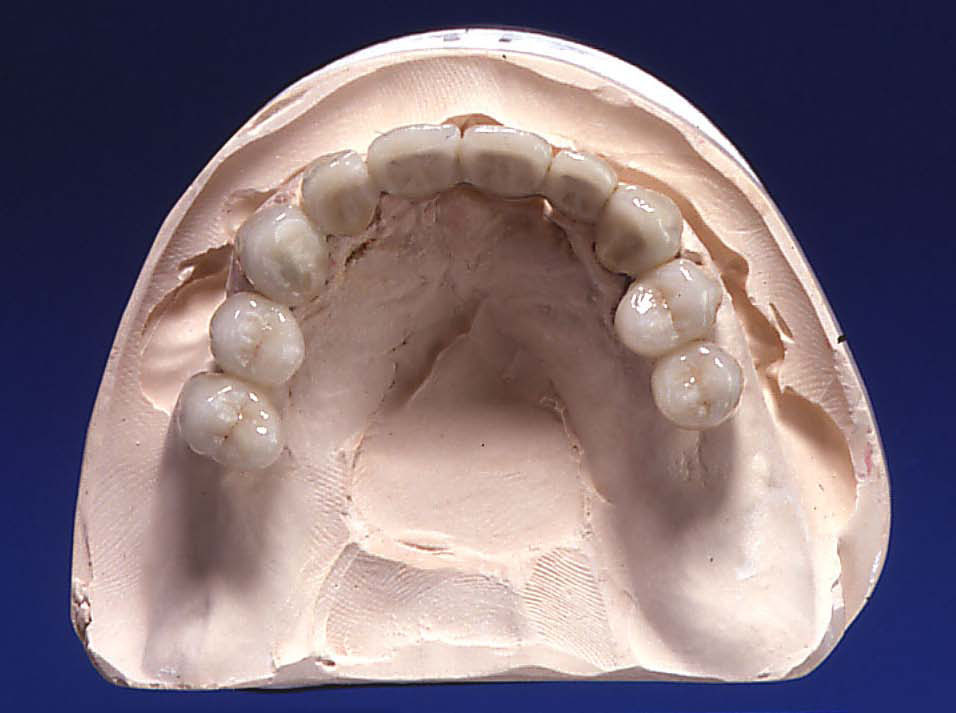
**Example of treatment B**.

Treatment B: Molars were not replaced. No prosthetic treatment was performed if the shortened dental arch was complete up to the second premolar. Any missing tooth up to the second premolar was replaced with tooth-borne bridges with or without cantilevers (Fig. 3).

In most cases, an appropriate pretreatment had to precede the final prosthetic phase. After completion of the pretreatment, the following conditions had to be achieved:

▪ all abutment teeth are either sound or had successfully undergone a periodontal treatment (pocket depth ≤ 4 mm, tooth mobility ≤ grade 2, mean plaque index ≤ grade 2 [24], bleeding on probing ≤ 25% of all probing sites)

▪ all teeth are either free of caries or restored adequately

The treatment was performed in compliance with the standard guidelines of a prosthetic textbook (20). If necessary, the opposing jaw was restored with fillings, crowns, bridges, or RDPs. In the opposing jaw, the missing teeth were replaced at least up to the first molar (treatment A) or up to the second premolar (treatment B) in order to achieve adequate occlusion.

All restorations were made according to a standardized operating procedure (SOP) given by the study protocol. Common high noble alloys and common dental porcelain materials were used for the fixed restorations; non-precious alloys were chosen for the frameworks of the removable denture. The local investigator supervised the treatment procedure and had to give his/her approval prior to the final insertion.

### Objectives

The specific objective of this trial was to gain reliable outcome data with regard to different treatment options in SDA cases considering a multidimensional understanding of health. The study focused on general factors as tooth loss and treatment failure as well as on psychic aspects and aspects related to the patients' quality of life. Specific dental and oral factors encompassed caries, periodontitis, temporomandibular disorders, and aesthetics. In addition, technical factors (performance of the incorporated medical devices) were included. The hypothesis was that the outcomes of treatments A and B would differ.

### Outcomes

The primary and secondary outcome measures are listed in Table 1. Tooth loss after prosthetic treatment was chosen as the primary outcome measure due to its high impact on the oral health. Any tooth loss regardless of the jaw was counted. Thus, two questionnaires - the OHIP "Oral Health Impact Profile" [25] and the Dworkin Index "Research Diagnostic Criteria for Temporomandibular Disorders, Part III Examination and History Data Collection" [26] - originally in the English language - were translated into German. Methodological aspects were taken into account by involving native speakers during translation and retranslation into German or English respectively.

Independent experts in the fields of periodontology, cariology and TMD performed the training and calibration of the local investigators and dentists responsible for treatment measures. During the first calibration meeting, videos demonstrating the clinical procedures were recorded and made available to all participating clinics. The information is also available on the study homepage in a password-protected area.

Both the dentist and the local investigator were trained to complete the data forms in a defined order to ensure data integrity. The clinical training and the calibration comprised the collection of the measurable biological parameters (pocket depths etc.) and the assessment and scoring of the softer variables related to the condition of the prostheses and aesthetics. For the quality assessment of the prosthetic appliances, the quality evaluation criteria of the California Dental Association (CDA) [27] were adapted to the specific needs of the trial (Table 2).

**Table 2:** Quality evaluation criteria according to the California Dental Association (CDA) [27]

	Rating scale	Criteria
**Satisfactory**	"Romeo"	Excellent clinical quality or performance
	
	"Sierra"	Acceptable clinical quality or performance

**Non-satisfactory**	"Tango"	Clinical quality or performance, which must be **repeated, replaced, repaired, or corrected **to avoid future damage for the patient
	
	"Victor"	Clinical quality or performance, which had to be **repeated, replaced, repaired, or corrected immediately **due to a damage occurring for the patient at that time

The analysis and the discussion of the pilot study results took place at an international workshop held in October 2001 in Dresden. Based on the results and experience gained during the pilot study, conclusions were drawn and changes in the study design were made.

The baseline examination was performed 4 to 8 weeks after the insertion of the prosthetic restorations by external investigators chosen randomly among the investigators of the other participating dental schools. The examinations had to be pooled to reduce the traveling costs. Approximately 10% of the patients were examined twice on a random basis by both the local and the external investigator. This applied also to all follow-up examinations.

### Sample size

The outcome measure "further tooth loss" required a sufficient number of patients in order to yield significant results in the primary efficacy analysis offering a high probability. In the course of the determination of the sample size, the number of the potentially eligible patients at the participating clinics per year was determined. Due to the small number of suitable patients, only a multi-site approach could provide an adequate number of participating patients. Furthermore, to ensure an impartial sampling, it was necessary to involve as many centers as possible.

Calculating the necessary number of patients to be treated, a two-year recruitment period and a five-year follow-up period were scheduled. During the follow-up, the expected tooth loss was presumed to reach 20% with treatment A and 5% with treatment B [13-15], and 5% of the patients were expected to switch the treatment. Applying the intention-to-treat principle, these patients are evaluated in the treatment group they were originally allocated to. Loss to follow-up of patients over time was assumed to follow an exponential distribution, the dropout rate adding up to 10% of recruited patients after 5 years. The maximum type I error of the applied two-sided significance test was set to 5%. The power was set to 75% in terms of detecting treatment differences of the above magnitude. According to the presumptions and requirements mentioned, the calculated number required amounted to a total of 70 patients per treatment group.

### Randomization - sequence generation

The randomization was performed using randomly permuted blocks for each dental clinic with stratification into two age groups: up to 50 years und above 50 years of age.

### Randomization - allocation concealment

The randomization concealment was warranted because the randomization was conducted centrally after the patient enrollment phase.

### Randomization - implementation

The data of any potentially eligible patient, who contacted one of the participating study centers, were documented during the screening process. Once a patient had given his/her informed consent, the data forms with clinical findings and treatment planning were sent to the treatment coordinator (TC). The TC checked the data regarding their conformity with the study protocol and sent a randomization request to the biometrical center. Finally, the TC forwarded the randomization results to the dental clinics.

### Blinding (masking)

Due to the visible discrepancy of the treatments, neither the dentist nor the patient could be blinded.

### Statistical methods

All statistical analyses are intended to be performed on the basis of the intention-to-treat principle, i.e. according to the patient's randomized treatment allocation regardless of any later protocol violations. In order to assure that treatment groups are balanced and provide structural equality, baseline patient characteristics will be compared between groups. For the primary outcome measure, Kaplan-Meier survival analyses will be performed. The Mantel-Cox log-rank test will be applied to test the null hypothesis of the equal progression of tooth loss in both treatment groups. This particular hypothesis test constitutes the primary study result and provides confirmatory statistical evidence. In order to quantify to what extent the risk to lose teeth differs between the treatment groups, the hazard ratio will be estimated on the basis of a univariate proportional hazards model. Beyond the primary analysis, analyses of prespecified secondary outcome measures will be performed. In further exploratory analyses, possible prognostic factors of tooth loss will be identified applying the multivariate Cox regression as well as classification and regression tree (CART) analyses.

Descriptive analyses generally comprise the calculation of location and scale statistics (mean and standard deviation) as well as rates of target events. Point estimates will be supplemented by 95% confidence intervals. Inductive statistical analyses will be performed using appropriate significance tests. In order to compare both treatment groups with respect to quantitative measures, the Mann-Whitney U test will be applied, and Fisher's exact test will be used for the qualitative factors. The statistical analyses will be performed by means of the software packages SPSS (Version 17 for Windows, SPSS Inc., Chicago, USA) and SAS (Version 9.2 for Windows, SAS Institute Inc., Cary, NC, USA).

### Enrollment, assessment, and follow-up

**Figure 4:**
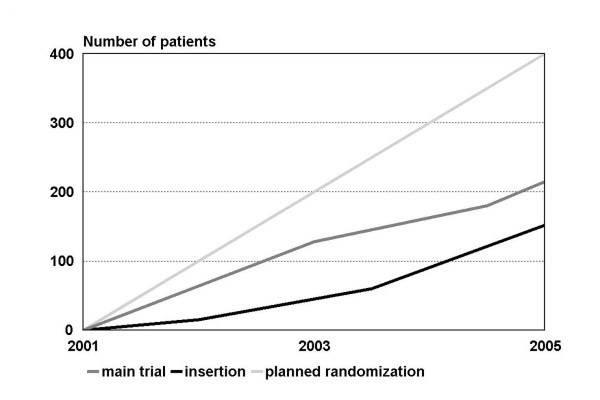
**Number of patients randomized and patients treated compared to the number originally scheduled**.

The enrollment for the pilot study was stopped at 36 patients, with 19 patients randomized for therapy A and 17 randomized for therapy B. Three patients dropped out after the randomization; in one of these cases, the treatment had been performed already. Two hundred and fifteen patients were enrolled in the main trial, with 109 patients randomized for treatment A and 106 for treatment B (Fig. 4). The follow-up appointments were scheduled at 4 to 8 weeks (baseline) and at 6, 12, 24, 36, 48, and 60 months after finishing the treatment (Table 3).

**Table 3:** Examinations

Examination Type	Examination time	Investigator
**Screening**	Every patient complying with the inclusion criteria regardless of his/her participation in the trial	Any dentist involved

**Pretreatment examination**	Before randomization, after giving informed consent	The dentist performing the treatment

**Insertion**	During insertion	Local investigator

**Recall after insertion**	1, 3 and 14 days after insertion	Local investigator

**Baseline**	4-8 weeks after insertion	Local investigator/external investigator (randomized selection)

**Recall**	6 months, 12 months, 2, 3, 4, 5 years after insertion	Local investigator/external investigator (randomized selection)

## Discussion

Traditionally, the treatment decisions in dentistry were based on empirical knowledge and in-vitro investigations of dental materials. During the last decade, clinical trials were carried out more frequently. However, high-level evidence is lacking regarding many treatment-related questions in dentistry due to:

(1) controversial opinions concerning feasible outcome measures in clinical studies (subjective, objective),

(2) long observation periods, particularly for the research in restorative dentistry,

(3) high treatment expenses and laboratory costs, and

(4) difficulties with regard to randomization and blinding respectively.

The aim of the majority of studies in restorative dentistry was to prove whether or not a treatment can be considered effective. The outcome measures used in dentistry usually focused on technical failures (restoration still present, survival rates) so far. Some studies considered biological aspects, for example periodontal condition and need of endodontic treatment. In recent years, the evaluation of patient-related factors and the oral health related quality of life have been gaining importance. While the majority of the previous studies had a retrospective design and relatively limited variable sets, this trial is aiming at a more comprehensive evaluation of the prosthetic treatment. This approach required the collection of a large-scale variable set. However, some of the very specific examinations used in the pilot study proved to be less meaningful than expected. Furthermore, it was difficult to train some of the respective diagnostic measures (Table 1). A multitude of the collected data resulted from the comprehensive evaluation of the prosthetic treatment methods and restoration types. This multitude of data could compromise the practicability. Considering the basic aim of the trial, it was vital to focus on those clinical findings that might lead to an explanation for the success or failure of a treatment concept, the onset of TMD, and patient satisfaction.

Discussions at the international symposium held after the recruitment phase of the pilot study revealed the treatment workflow as further cause for the limited number of patients recruited. The pilot study protocol demanded a completed pretreatment (i.e. caries, periodontitis) before randomization. The pretreatment sometimes required the removal of existing restorations without being able to give the patient a clear treatment perspective. Therefore, the treatment workflow was changed to randomization at an earlier stage.

Clinical trials on the long-term outcomes of prosthetic treatments are extremely costly and time-consuming. The observation periods required are long. The complexity of the prosthetic treatment measures and the common allocation practice with regard to the differential indication of fixed versus removable restorations [28] turn the randomized trial design into the only option feasible.

The most important confounders in prosthetic treatment studies are the dentists themselves, the applied treatment principles, and the treatment environment. The multi-center approach involving almost half of the German dental schools and the extensive training of the dentists are considered appropriate to reduce this bias and appear to be suitable to deliver results that are representative for the basic conditions of German dental-schools.

The main inclusion criteria define a certain topography where all molars are missing in one jaw and at least both canines and one premolar are present on each side (Fig. 1). This is an appropriate condition to study the differential indication between removable (treatment A) and fixed (treatment B) restorations and an ideal field to gather comparable data on the clinical performance of these fundamentally differing treatment concepts. However, the number of cases was lower than expected because of problems during the patient recruitment phase and an overestimation of the number of eligible subjects (Fig. 4). Due to methodological reasons, the inclusion criteria had to be chosen in a manner that the two fundamentally differing treatment concepts could be applied with similar chances of success and therefore be comparable accordingly. This applies to the suitable dental status as well as to the exclusion of conditions that might compromise one of the treatment concepts, for example malocclusion in treatment B. Even the multi-site approach, numerous advertisements in local newspapers, and further measures to support the recruitment proved to be insufficient to raise the number of patients targeted.

Following the holistic approach, clinical findings were applied that are commonly used in the daily practice. Only few diagnostic measures applied in clinical dentistry have been validated (e.g. periodontal probing depth) including the possibility to gather clinical data in a reproducible way and, thus, allow the calibration of the clinical investigators. In general, surrogate parameters as caries and attachment loss have widely been used in prosthetic treatment studies. They are suitable to deliver relatively early results and were, therefore, included as secondary outcome measures. However, tooth loss as the irreversible sequel of the main dental diseases has a high reliability and a high impact on health and was, therefore, chosen as a very viable indicator of oral health.

The study can be considered as one of the most comprehensive randomized trials in prosthetic dentistry. The design with its basic principles established the basis for clinical research in restorative dentistry on a contemporary level. Considering the basic aim of the trial, it was vital to focus on those clinical findings that might offer an explanation for the success or failure of a treatment concept, the occurrence of TMD, and, not least important, patient satisfaction.

## List of abbreviations used

ASA: American Society of Anesthesiologists; CART: Classification and Regression Trees; CDA: California Dental Association; DFG: Deutsche Forschungsgemeinschaft (German Research Foundation); EN: Europäische Norm (European Standard); ISO: International Organization for Standardization; OHIP: Oral Health Impact Profile; RaSDA: The Randomized Shortened Dental Arch Study; RDP: Removable Dental Prosthesis; RDPs: Removable Dental Prostheses; SDA: Shortened Dental Arch; SOP: Standardized Operating Procedure; TC: Treatment Coordinator; TMD: Temporomandibular Disorders.

## Competing interests

The authors declare that they have no competing interests.

## Authors' contributions

RL made substantial contributions to the conception and design and was involved in drafting the manuscript. MW is the principal investigator. BM made substantial contributions to the acquisition of data. MW and BM were involved in drafting the manuscript. AH and JG accomplished the statistical consultancy and computation, JG was also involved in drafting the manuscript. HA, EB, PD, IG, SH, and FJ made substantial contributions to the acquisition of data. WH, MK, TM, PP, HS, and MW made substantial contributions to the conception and design. GH made substantial contributions to the acquisition of data and to the analysis and interpretation of data. BW made substantial contributions to the conception and design and provided the data base to enter the data of the study into SPSS. HA, EB, PD, IG, AH, SH, WH, FJ, MK, TM, PP, and HS were involved in critically revising the manuscript. All authors read and approved the final manuscript.
